# Differential Mobility Spectrometry-Tandem Mass Spectrometry with Multiple Ion Monitoring Coupled with in Source-Collision Induced Dissociation: A New Strategy for the Quantitative Analysis of Pharmaceutical Polymer Excipients in Rat Plasma

**DOI:** 10.3390/molecules28124782

**Published:** 2023-06-15

**Authors:** Yuyao Zhang, Zhi Zhang, Yingze Liu, Deqi Cai, Jingkai Gu, Dong Sun

**Affiliations:** 1 Research Center for Drug Metabolism, School of Life Science, Jilin University, Changchun 130012, China; 2 State Key Laboratory of Supramolecular Structure and Materials, College of Chemistry, Jilin University, Changchun 130012, China

**Keywords:** differential mobility spectrometry, multiple ion monitoring, polylactic acids, quantitative analysis, pharmaceutical polymer excipients

## Abstract

Polylactic acids (PLAs) are synthetic polymers composed of repeating lactic acid subunits. For their good biocompatibility, PLAs have been approved and widely applied as pharmaceutical excipients and scaffold materials. Liquid chromatography-tandem mass spectrometry is a powerful analytical tool not only for pharmaceutical ingredients but also for pharmaceutical excipients. However, the characterization of PLAs presents particular problems for mass spectrometry techniques. In addition to their high molecular weights and wide polydispersity, multiple charging and various adductions are intrinsic features of electrospray ionization. In the present study, a strategy combining of differential mobility spectrometry (DMS), multiple ion monitoring (MIM) and in-source collision-induced dissociation (in source-CID) has been developed and applied to the characterization and quantitation of PLAs in rat plasma. First, PLAs will be fragmented into characteristic fragment ions under high declustering potential in the ionization source. The specific fragment ions are then screened twice by quadrupoles to ensure a high signal intensity and low interference for mass spectrometry detection. Subsequently, DMS technique has been applied to further reduce the background noise. The appropriately chosen surrogate specific precursor ions could be utilized for the qualitative and quantitative analysis of PLAs, which provided results with the advantages of low endogenous interference, sufficient sensitivity and selectivity for bioassay. The linearity of the method was evaluated over the concentration range 3–100 μg/mL (r^2^ = 0.996) for PLA 20,000. The LC-DMS-MIM coupled with in source-CID strategy may contribute to the pharmaceutical studies of PLAs and the possible prospects of other pharmaceutical excipients.

## 1. Introduction

Nano-drug delivery system (NDDS) has been developed rapidly in recent years. It may improve the pharmacokinetics and pharmacodynamics properties of active pharmaceutical ingredients (APIs), especially for the APIs with narrow therapeutic window and pervasive cytotoxicity. NDDS is aimed to reduce premature degradation, improve drug uptake, sustain drug concentrations within the therapeutic window, as well as to reduce side effects [1]. The most investigated application scenario of NDDS is cancer therapy, in terms of increasing solubility and bioavailability, prolonging the circulation time via passive or active targeting and increasing the accumulation of APIs in tumor tissue [2]. To support the development of novel and effective NDDS, various synthetic polymeric excipients have been investigated and evaluated. The quantitative analysis of these polymeric excipients is therefore of pharmaceutical significant.

PLA is an FDA-approved polymeric excipient for human usage, which has been widely used for nanoparticles and scaffolds materials because of its good biocompatibility [3,4]. PLA nanoparticle formulations such as liposomes, dendrimer or micelles exhibited good targeting and low systemic toxicity, and turned to be an attractive formulation in term of antitumor drug delivery [5,6]. Polymeric excipients were usually considered to be biologic inactive, whose pharmaceutical properties have been underestimated. With the deepening of understanding, more and more biological effects caused by pharmaceutical excipients have been discovered, e.g., immunoreaction, anaphylactic reaction and drug-drug interaction. Recent studies indicated that PLAs with slow metabolic and clearing rates may accumulate in liver and kidney [7,8], adverse interactions between PLA excipients and delivered APIs have been disclosed [9,10,11]. A full characterization of the in vivo fate and pharmacokinetic behavior of PLAs after administration is indispensable for interpreting or estimating the excipient-related toxicity. Therefore, there is an urgent need for a reliable and robust analytical method for the quantitation of PLAs in biological samples.

Currently, strategies such as nuclear magnetic resonance (NMR), permeation chromatography (GPC) and matrix-assisted laser desorption ionization-time of flight (MALDI-TOF) MS have been applied to the characterization of PLA [12,13,14,15]. While, NMR is hard to be applied to the analysis of PLA in complicated biomatrix. GPC and MALDI-TOF were also interfered by endogenous substance which had the similar molecular weight (MW) to analyte in biological sample quantification process. Besides, MALDI-TOF was reported that had deviation of detector response while analyzing polydispersity polymers [16]. This study indicates that the high-mass components are generally underrepresented with respect to the lower mass components. The aforementioned techniques are insufficient in terms of sensitivity and specificity, which are not suitable for the analysis with complex matrices such as plasma and tissue homogenates, because of matrix interference. Enzyme-linked immune sorbent assays (ELISA) have been used for polymer analysis [17,18,19]. However, shortcomings such as cross-reactivity, complicated operation restrict the application of ELISA in the pharmaceutical analysis. Most literatures reported polymer analytes quantified by ELISA were polyethylene glycol (PEG) and PEGylated derivates. Design and synthesis of antibody to determine other different kinds of polymers also incurs additional workload and extra cost. Fluorescent labeling and radiolabeling were also applied to analyze pharmaceutical polymers [20,21]. They belong to indirect analysis methods which detected analytes by the fluorescence signal or radioactive signal of the labeling group. With the introduction of labeling group, the pharmacokinetic behavior of analyte will be influenced because of the change of chemical structure. It was reported that even if using ^3^H instead of ^1^H would lead to a changed pharmacokinetics of drugs [22]. Besides, the shedding of labeling group would cause a false positive result and the radiolabeling group is potentially harmful to human body and environment.

Liquid chromatography-tandem mass spectrometry (LC-MS/MS) has been widely applied to analyze biological samples due to its excellent selectivity and sensitivity. Osaka et al. have developed a quantitative determination method for cyclic polylactic acid hep-tamer in serum based on LC-MS/MS [23]. However, the method cannot be expanded to PLA. Unlike small molecules, polymer MW is defined as a distribution rather than a cer-tain number. The polydispered MW generates series of precursor ions by electrospray ionization (ESI). Therefore, it is difficult to select all of the precursor ions of PLA by quad-rupole (Q) for a comprehensive analysis, which is limited by the scanning speed of MS. Gong et al. recognized the problem and developed an in source-CID strategy to quantify PEG and PEGylated proteins in animal tissue [24], in that all of the PEG-related precursor ions were to be dissociated into ethylene glycol (EG) oligomers in the ion source under high declustering potential (DP). The original function of DP is to decluster the ion clusters produced in ionization process. Excessive DP will lead to in source-CID, which decreases sensitivity of analytical method and in source-CID should be avoid during small molecules analysis process. Nevertheless, this in source-CID phenomenon contributes to polymer quantitation by LC-MS/MS. The uncountable precursor ions of polymeric analyte can be therefore reduced to limited specific EG-species within source-CID, which will be selected by Q1 as the surrogate precursor ions and fragmented at Q2. The product ions of the surrogate precursor ions are to be monitored by Q3 This strategy is known as multiple reaction monitoring (MRM) combined with in source-CID, which has been successfully applied to the quantitation of various polymers [25,26,27,28].

When applied to the analysis of PLA 20,000, the in source-CID/MRM strategy has en-countered a new obstacle, namely poor fragment efficiency. Its specific surrogate precursor ions obtained by in source-CID are mainly sodium adduct ions [M + Na]^+^. When comparing with protonated species, sodium adduct ions are stubborn to be fragmented. No suitable fragmentation reaction of the PLA could be utilized by the MRM mode, even under high collision energy (CE). Subsequently, MIM technique coupled with in source-CID has been tested in order to address this problem. Under MIM scanning mode, the selected ions by Q1 and Q3 are identical, Q2 only plays a role of transmission. Utilizing LC-MIM coupled with in source-CID, intensive and stable fragment ions of PLA has been identified. However, this approach has its instinct shortcoming, namely poor specificity. Lacking of the ion transformation in Q2, the interferences in the matrix and background were significant [29]. Additional compensatory measures should be taken to decrease the interference and background noise to achieve the quantitation of PLA 20,000.

Differential mobility spectrometry (DMS) is a newly emerged technology, which can be deemed as additional separating procedure according to the different mobilities of ions under an asymmetric oscillating electric field [30,31,32,33,34]. By applying a characteristic separation voltage (SV) in combination with a specific compensation voltage (COV) in DMS device, merely the analyte of interest with matched mobility is allowed passing through. All the interferences, even the geometric isomer of the analyte, will ram into the electrodes eventually and cannot reach Q1 eventually. As a result, chemical background noise and matrix interference could be significantly reduced. In the previous study of our group, combining DMS, LC-MIM and in source-CID could be achieved by combining DMS, LC-MIM and in source-CID [35]. However, if the strategy could be applied to the quantitation of polydispersity polymer is still doubtful. In the present, we optimized LC-DMS-MIM/in source-CID strategy for the characterization and quantitation of polydisperse PLA 20,000 in rat plasma. This strategy might be applied to reveal the in vivo pharmacokinetics of various polymeric excipients and contribute to the development of novel NDDS.

## 2. Results and Discussions

### 2.1. MS Analysis of PLAs

#### 2.1.1. Fragmentation of PLA 20,000 by in Source-CID

**Figure 1:**
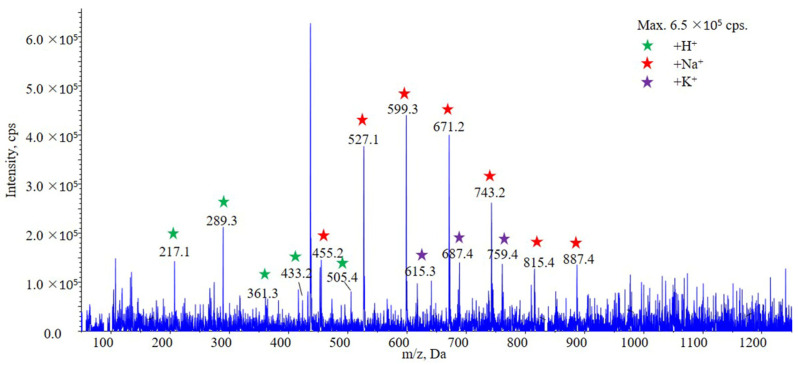
MS scanning result for PLA 20,000 using in source-CID. Different series ions were observed, including [M + H]^+^ series ions (*m*/*z* 217.1, 289.3, 361.3, 433.2 and 505.4), [M + Na]^+^ series ions (*m*/*z* 455.2, 527.1, 599.3, 671.2, 743.2, 815.4 and 887.4) and [M + K]^+^ (*m*/*z* 615.3, 687.4 and 759.4) were observed.

PLA 20,000 chains would undergo CID by the energy of DP in the ionization source. With the increasing of DP, series of surrogate precursor ions with different lactic acid (LA) units (72 Da) were observed viz. proton, sodium and potassium adduct ions (Figure 1). The prominent thereof were sodium adduct ions [72 n + Na]^+^ (e.g., [7 LA + Na]^+^ at *m*/*z* 527.2, [8 LA + Na]^+^ at *m*/*z* 599.3, [9 LA + Na]^+^ at *m*/*z* 671.2, etc.), which had strong and stable signal intensity. The mass response of [M + H]^+^ and [M + K]^+^ series were relatively low. In brief, the numerous precursor ions of PLA have been reduced to countable few by in source-CID. With the help of these surrogate precursor ions, quantitation of PLA seems to be feasible. 

#### 2.1.2. Fragmentation Efficiency of the Sodium Adduct Ions [M + Na]^+^

**Figure 2:**
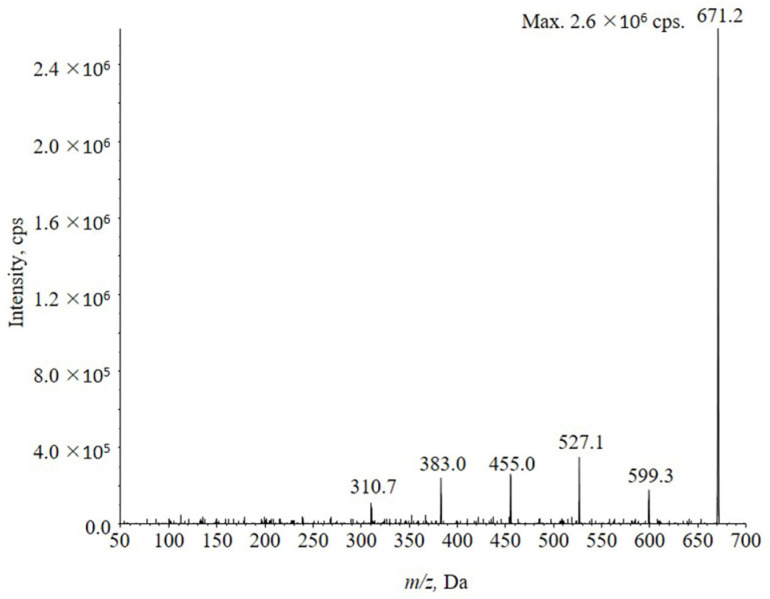
MS2 scanning result for PLA 20,000 under CE 80 eV. Precursor ion *m*/*z* 671.2 still had high MS intensity and fragment ions produced by *m*/*z* 671.2 was not stable with low intensity under such fragment condition. The result showed the poor fragmentation efficiency of PLA 20,000.

The sodium adduct ions [M + Na]^+^ can be hardly fragmented in Q2 even under high CE. For example, the sodium adduct ion at m/z 671.2 gave only a few product ions under CE 80 V, namely *m*/*z* 310.7, 383.0, 455.0, 527.1 and 599.3. (Figure 2). The mass difference between these species was 72 Da, which was one LA unit to confirm they were product ion of precursor ion *m*/*z* 671.2. The signal intensity of these product ions was faint, meanwhile the intensity of *m*/*z* 671.2 was dominant. The [M + Na]^+^ was stubborn under high CE.

Traditional MRM mode for quantitation has high selectivity and sensitivity which is suitable to analyze in vivo samples. However, it requires high fragmentation efficiency to produce high intensity and stable product ion from precursor ion. As a result of this section, the poor fragmentation efficiency of sodium adduct ions severely restricted the determination of PLAs, since no suitable fragmentation reaction could be utilized for MRM. Considering the signal intensity and stability of [M + Na]^+^, the MIM scanning mode seems appropriate for conducting instead of fragmenting the ions with poor fragmentation efficiency, because there is no need of ion transformation.

### 2.2. Application of DMS

The strategy of MIM combined with in source-CID showed a great potential for the analysis of PLAs. The technique obstacle was the serious matrix interference and high background noise because of the lack of selectivity. Therefore, there is a need to introduce a new separation dimension to distinguish PLA from in vivo interference.

DMS is a new separation method which is compatible with LC-MS system. The separation principle of DMS is the differential mobility between different ions. Different ions in a certain electric field would generate differential mobility because of their cross-sectional area and charge. It means that even two compounds share the same *m*/*z*, DMS could separate them due to their structure difference and charge difference. DMS device is placed prior to Q1 quadrupole of MS. Ions pass through between two parallel electrodes with the help of transport gas. SV between two parallel electrodes is an asymmetric radio frequency waveform, which forces ions to move radially. If there was no additional voltage, ions would strike to an electrode and trap in DMS device, which could not transport to MS. At the same time, a direct voltage named COV is applied to electrodes and pulls back the ions towards to the electrodes. Ions that matched the COV could pass through the gap between electrodes against SV and get into MS eventually, while other ions strike to electrodes. By adjusting SV and COV, target ions can be separated from interference compounds which increase the selectivity of analytical method. Application of DMS would cause a decrease of absolute intensity of analyte while increasing the signal-to-noise ratio (S/N). Therefore, the optimization of DMS parameters is an especially crucial process to obtain the highest MS intensity and ensure the limit of quantitation of analyte.

**Figure 3:**
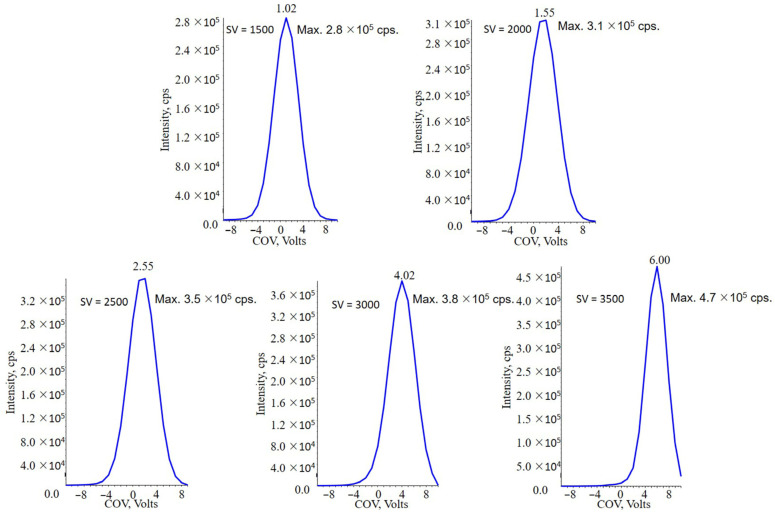
Optimization of SV and COV of PLA 20,000. The result showed under the conditions of SV 3500 V and COV 6.00 V obtained the highest MS intensity.

**Figure 4:**
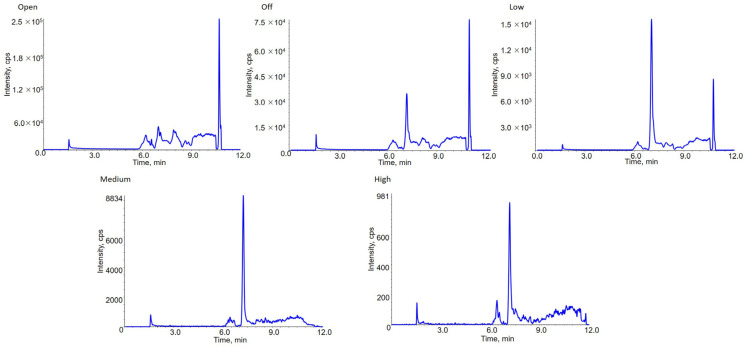
Optimization of DR of PLA 20,000. Figures showed chromatograms under different DR settings and at “medium” setting obtained the highest S/N of chromatographic peak.

DMS has been coupled with MIM as a compensatory selection condition. DMS pa-rameters were optimized by ramping COV under different SV and infusing PLA 20,000 sample via the syringe pump. SV of 1500, 2000, 2500, 3000 and 3500 V have been investi-gated. Each SV matched a specific COV value to reach the highest MS intensity. The opti-mized SV and COV of PLA 20,000 were 3500 V and 6.00 V respectively (Figure 3). DMS resolution enhancement (DR) is another important parameter in the system. It is able to adjust the S/N of target compound. The influences of DR settings at open (0 psi), off (9.9 psi), low (22 psi), medium (34 psi) and high (39 psi) have been compared and evaluated. Results showed that with the increasing DR settings, the intensity of PLA 20,000 and background noise decreased, but the S/N was improved from “open”setting to “medium”setting. At “high”setting, the S/N of PLA 20,000 started to get lower. As a result, the setting “medium” provided the highest S/N ratio (Figure 4). In addition, the influence of the temperature of DMS (DT) at low (150 °C), medium (225 °C) and high (300 °C) has been also investigated. The MS response at low DT was optimal.

### 2.3. Comparation between LC-MRM, LC-MIM and LC-DMS-MIM

**Figure 5:**
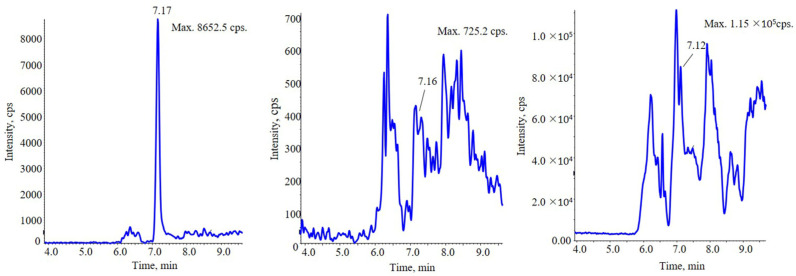
Chromatography of PLA 20,000 at LLOQ concentration (3 μg/mL) under LC-DMS-MIM, LC-MRM and LC-MIM scanning modes. With the comparation between these modes, LC-DMS-MIM showed the best separation effect of PLA 20,000 from interference and background noise in plasma matrix.

To illustrate the advantages of the strategy in the present work, comparison between the results of PLA 20,000 at the lower limit of quantitation (LLOQ) by LC-MRM, LC-MIM and LC-DMS-MIM has been conducted (Figure 5). For LC-MRM, the fragmentation reaction at *m*/*z* 671.2 → 383.2 has selected in spite of poor fragmentation efficiency. No obvious peak of analytes could be observed. For LC-MIM merely, *m*/*z* 671.2 → 671.2 was monitored. The background noise or matrix interference was nonnegligible. The peak of interest analytes was submerged. In stark contrast, LC-DMS-MIM showed an enhanced specificity as well as S/N ratio. 

**Figure 6:**
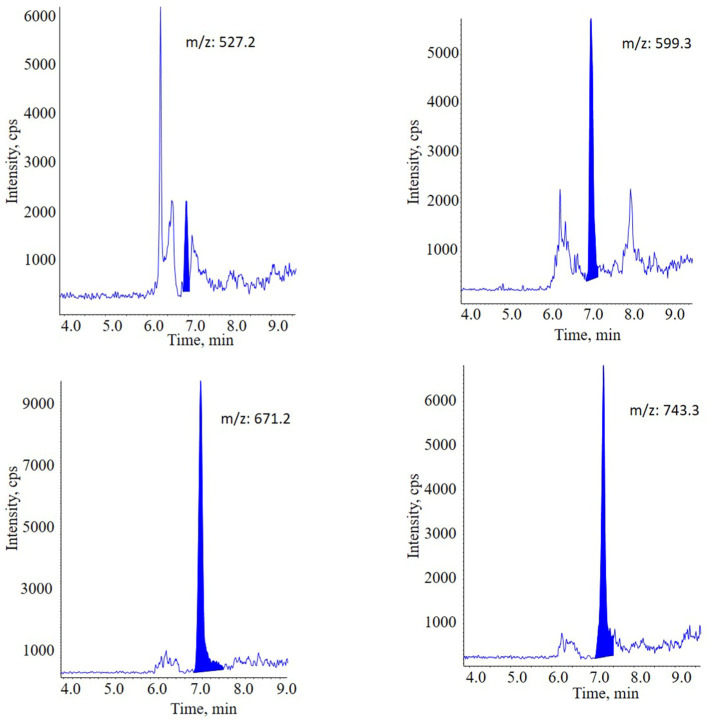
Chromatography of different ions of PLA 20,000 under LC-DMS-MIM mode with in source-CID, including [7 LA + Na]^+^, [8 LA + Na]^+^, [9 LA + Na]^+^ and [10 LA + Na]^+^. Among these four surrogate precursor ions, [9 LA + Na] (*m*/*z* = 671.2) showed the highest intensity and S/N.

However, the separation ability of DMS is limited. It is still necessary to screen out appropriate surrogate precursor ions with minimum interference. A series of specific sur-rogate precursor ions were investigated, and each specific ion was interfered with matri-ces and endogenous substances at varying degrees. As the result of balancing between peak intensity and interference, [9 LA + Na]^+^ (*m*/*z* 671.2) was chosen as the surrogate quantitative ions for PLA 20,000 (Figure 6). Generally speaking, the screening of surrogate quantitative ions is critical for this strategy.

### 2.4. Optimization of LC Condition

A PLRP 1000 Å column (150 × 2.1 mm, 8 μm, Agilent) exhibited desirable separation ability for PLAs. After optimization, it was found that a symmetrical chromatographic peak with minimum carryover can be achieved with the water/acetonitrile eluent system at 50 °C column temperature.

### 2.5. Sample Preparation

An extraction method with high recovery rate is the key step for the accurate characterization and quantitation, especially for the polymeric sample with a low concentration in biological samples. During the method development, liquid-liquid extraction (LLE) and solid-phase extraction (SPE) and protein precipitation (PPT) have been evaluated and compared. Only PPT exhibited a rate of recovery over 50%. PPT with acetonitrile, methanol and isopropanol used as precipitated reagent at different ratios were investigated. The result indicated that acetonitrile provided a better precipitation effect. A desired rate of recovery and minimum matrix effect have been achieved at the acetonitrile/plasma volume ratio of 5:1, meanwhile lower sample volume is favorable for achieving a lower quantitation limit.

### 2.6. Assay Validation

**Figure 7:**
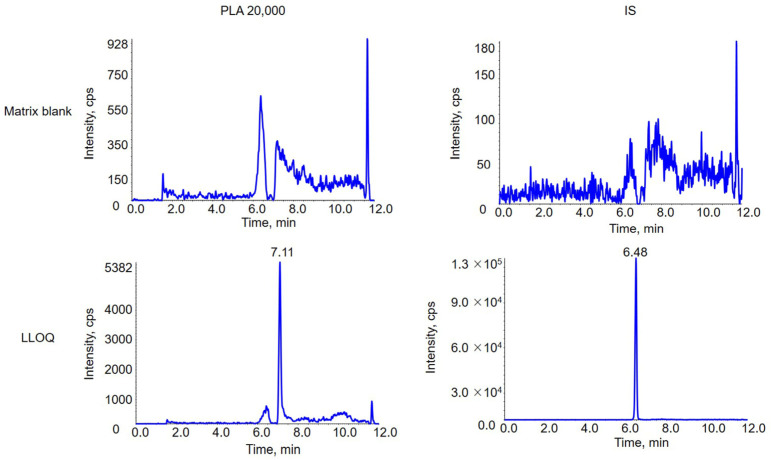
Chromatography of matrix blank and LLOQ samples of PLA 20,000 and IS.

No significant endogenous interference in the biomatrix blank sample with the identical retention time of PLA and IS has been observed by the LC-DMS-MIM/in source-CID method in matrix blank sample (Figure 7). Evaluation of linearity showed the assay was linear over range of 3–100 μg/mL (r^2^ = 0.996) for PLA 20,000. Intra- and inter-day precision and accuracy were all within accepted values of ±15%. Corresponding data was listed in Table 1. The result indicated that the established method was robust and accurate. Matrix effects of PLAs have been evaluated with QCs at low, medium and high concentrations, namely 91.2 ± 5.4%, 92.8 ± 6.6% and 97.5 ± 7.8% of PLA 20,000. Corresponding rate of recoveries were 101.2 ± 8.8%, 103.4 ± 4.5% and 99.4 ± 8.6% respectively. The established method provides a low matrix effect and good recovery, which ensure the reproducibility of the accuracy of the method during the sample preparation and analysis processes. The mean peak area at retention time of carryover blank sample direct after the ULOQ samples was less than 20% peak area of the LLOQ sample (9.39 %). There is no significant carryover effect. Stability results were all within accepted values of ± 15 %, which showed that PLA 20,000 was stable at −80 °C for 2 weeks, room temperature for 3 h after sample preparation, autosampler for 12 h and after three freeze/thaw cycles. Detailed stability data was given in Table 2.

## 3. Materials and Methods

### 3.1. Chemicals and Reagents

PLA 20,000 was purchased from Sigma-Aldrich Co. (St. Louis, MO, USA). PEG 2000 for use as internal standard (IS) was provided by Applied Chemistry Institute of Changchun (Changchun, China). HPLC grade acetonitrile was purchased from Fisher Scientific (Fair Lawn, NJ, USA). Distilled water was obtained from Watsons (Beijing, China).

### 3.2. LC Condition

Chromatography was conduct on Agilent 1100 series LC system (Agilent Technology, Waldbronn, Germany), a PLRP-S 1000 Å (150 × 2.1 mm, 8 μm) column maintained at 50 °C with a flow rate of 0.3 mL/min and the injection volume was 10 μL. The mobile phase B was acetonitrile and A was distilled water. The gradient elution of PLAs was as follow: 0–2 min 20% B, 2–3 min 20–95% B, 3–8 min 95% B, 8–8.1 min 95–20% B, 8.1–12 min 20% B.

### 3.3. DMS and MS Conditions

A Qtrap 6500 mass spectrometer equipped with DMS device system (SCIEX, Concord, ON, Canada) was used for detecting. The system was operated in the positive ESI source. Detailed optimized MS and DMS parameters of PLA 20,000 and IS are shown in Table 3.

### 3.4. Preparation of Calibration Standards and Quanlity Control (QC)

PLA standard solution (2 mg/mL) was prepared in acetonitrile. Calibration standards were all prepared by diluting PLA standard solution with blank rat plasma to final concentration of 3, 5, 10, 20, 30, 50 and 100 μg/mL. QC samples were prepared at the same way to final concentration of 4, 24 and 90 μg/mL for PLA 20,000. PEG 2000 was dissolved with water and diluted to 10 μg/mL with acetonitrile-water (1:1, *v*/*v*) as IS solution. All solutions and samples were stored at −20 °C before use.

### 3.5. Sample Preparation

50 μL plasma sample mixed with 50 μL IS and 250 μL acetonitrile, were vortexing sharply for 1 min and centrifuging at 13,000 rpm for 5 min at 4 °C. 10 μL of the supernatant was injected into LC-DMS-MIM coupled with in source-CID system for analysis.

### 3.6. Assay Validation

The current LC-DMS-MIM/source-CID method was validated in accordance with FDA for regulated LC-MS bioanalysis. Specificity was evaluated by analysis of blank plasma samples from 6 rats. Linearity were assessed for a range of 3 to 100 μg/mL for PLA 20,000 by three separate calibration curves. LLOQ was defined as the lowest concentration on the calibration curve. Six replicates of LLOQ and low, medium and high QCs samples on three different days were used to evaluate intra- and inter-day precision and accuracy. The assessment of recovery and matrix effect was investigated at three concentration levels viz. low, medium and high QCs. In addition, potential carryover has been evaluated by blank plasma samples after the analysis of a sample with a concentration at the upper limit of quantitation (ULOQ). The carryover blank samples were analysed for 6 times. Stability of PLA 20,000 were evaluated in low and high QCs in triplicate stored at −80 °C for 2 weeks, room temperature for 3 h after sample preparation, autosampler for 12 h and after three freeze/thaw cycles.

## 4. Conclusions

In the present study, an analytical strategy based on LC-DMS-MIM combined with in source-CID for the quantitation of PLAs has been developed and evaluated. PLA 20,000 in rat plasma has been characterized and quantified over the concentration range 3-100 μg/mL. The strategy demonstrates advantages in specificity and sensitivity as well as valuable possible applications in the analysis of polymeric pharmaceutical excipients with limited fragmentation efficiency in traditional mass spectrometry methods. Furthermore, revealing the in vivo pharmacokinetics of polymeric excipients might greatly benefit the development of NDDS and novel pharmaceutical excipients.

## Figures and Tables

**Table 1:** Intra- and inter-day accuracy and precision results of PLA 20,000 (*n* = 6).

Spiked Concentration (/μg·mL^−1^)	Day	Intra-Day Accuracy (DEV %)	Intra-Day Precision (CV %)	Inter-Day Accuracy (DEV %)	Inter-Day Precision (CV %)
3.00 (LLOQ)	1	−0.94	5.31	−2.46	5.86
	2	−3.56	6.65		
	3	−2.89	6.34		
4.00	1	4.58	3.88	6.18	5.24
	2	4.38	6.61		
	3	9.58	4.12		
24.00	1	11.39	4.02	9.07	4.28
	2	10.42	2.93		
	3	5.42	4.12		
90.00	1	4.65	3.68	3.98	3.52
	2	5.24	2.13		
	3	2.06	4.25		

**Table 2:** Stability of PLA 20,000 under different storage conditions (mean ± SD, *n* = 3).

Storage Conditions	Low QC Samples (%)	High QC Samples (%)
Long terms (−80 °C, 14 days)	94.4 ± 3.6	94.1 ± 4.5
Three freeze-thaw cycles	92.7 ± 6.0	95.5 ± 3.7
Room temperature after sample preparation (25 °C, 3 h)	93.4 ± 4.2	96.0 ± 1.8
Autosampler (8 °C, 12 h)	100.6 ± 5.7	101.5 ± 5.3

**Table 3:** Optimum parameters for the determination of PLA 20,000 and PEG 2000 (IS) in rat plasma using LC-DMS-MIM with in source-CID.

Parameters	PLA 20,000	PEG 2000 (IS)
Ionspray voltage (V)	5500	5500
Curtain gas (N_2_, psi)	25	25
Source temperature (°C)	500	500
Nebulizer gas (N_2_, psi)	45	45
Heater gas (N_2_, psi)	45	45
*m*/*z* transition	671.2 → 671.2	133.0 → 89.1
Declustering potential (V)	180	210
Collision energy (eV)	20	11
DMS temperature (°C)	150	150
DMS resolution enhancement	Medium	Medium
DMS separation voltage (V)	3500	3500
DMS compensation voltage (V)	6	8

## Data Availability

Not applicable.
